# Quantification of training load across two competitive seasons in elite senior and youth male soccer players from an English Premiership club

**DOI:** 10.5114/biolsport.2023.126667

**Published:** 2023-06-12

**Authors:** Ryland Morgans, Dave Rhodes, Jose Teixeira, Toni Modric, Sime Versic, Rafael Oliveira

**Affiliations:** 1 Football Performance Hub, University of Central Lancashire, Preston PR1 2HE, UK; 2 Research Centre in Sports Sciences, Health and Human Development, 5001–801 Vila Real, Portugal; 3 Department of Sport and Physical Education, Polytechnic Institute of Bragança, 5300–253 Bragança, Portugal; 4 Polytechnic Institute of Guarda, 6300–559 Guarda, Portugal; 5 Faculty of Kinesiology, University of Split, Split, Croatia; 6 High Performance Sport Center, Croatian Olympic Committee, Zagreb, Croatia; 7 Sports Science School of Rio Maior–Polytechnic Institute of Santarém, 2040–413 Rio Maior, Portugal; 8 Life Quality Research Centre, 2040–413 Rio Maior, Portugal

**Keywords:** Global Positioning System, Performance, Training adaptations, Elite European soccer players, Competitive level, Football

## Abstract

This study aimed to compare the daily training load (TL) in first-team and U-18 soccer players from an English Premiership club. 36 first-team (age 23.2 ± 5.9 years, weight 75.2 ± 8.1 kg, height 1.83 ± 0.06 m), and 22 U-18 players (age 17.5 ± 1.1 years, weight 71.1 ± 8.2 kg, height 1.78 ± 0.08 m) participated. GPS metrics were measured during all pitch training sessions throughout the 2020–21 and 2021–22 seasons. Linear mixed-effect model analyses revealed that, irrespective of training day, U-18 players covered greater total and explosive distance than first-team players, and performed a higher number of accelerations and decelerations, whereas first-team players covered greater sprint distance. Irrespective of the team, all examined variables were greater at match-day (MD)-3, while the number of accelerations and decelerations were higher at MD-4. Significant team-by-training day interactions revealed that U-18 players covered greater total and high-intensity distances than first-team players at MD-4, MD-2, and MD-1, whereas first-team players covered greater total and high-intensity distances at MD-3. Sprint distance was greater for first-team players at MD-3 and MD-4, while explosive distance was greater for U-18 players at MD-2. Also, U-18 players performed a higher number of accelerations than first-team players at MD-3 and MD-2, and a higher number of decelerations at MD-4. The present results provide novel information on TL patterns in English Premiership soccer and contribute to understanding how training methods to physically develop players are implemented in different countries and leagues.

## INTRODUCTION

Soccer is a sport that is intermittent in nature with short periods of high-intensity activity separated by longer periods of low-intensity actions [1]. Knowledge of these characteristics are vitally important to adequately prepare players for the demands of match-play and have implications for training prescription [2]. The occurrence of these high-intensity activities during match-play has increased over the last few decades [3, 4], thus driving the development of specific training to allow players to cope with these ever-evolving demands. The progress of these physical demands has aligned with advancements in technology which have enhanced the ability of practitioners to quantify these aspects of physical performance during soccer training and, consequently, it allowed better training prescription, load adjustment and thus adequate preparation of players for match-play [5].

The increasement in physical demands and availability of technology has led to a rise in the popularity of quantifying player activities during training on a daily basis [6]. The quantification of physical training demands is commonly referred as training load (TL) [7]. Training load can be subdivided into external and internal dimensions. External load refers to the locomotive profile of individual players during training, while internal load refers to the individual physiological response to external load [8, 9]. In elite contemporary soccer clubs, both external and internal load are monitored respectively using various microelectromechanical systems and heart-rate telemetry systems to quantify the overall load placed on players during training and match-play [10].

The quantification of external and internal intensity under differing conditions and across various sessions and seasonal periods (pre-, in-season mesocycles) have been systematic reviewed and employed various monitoring methods such as total distance, high-speed running, sprinting, accelerations and decelerations in professional [11] and youth male soccer players [12]. However, the same study [11] highlighted a limitation that the varying training made it difficult to provide benchmark values for the key external and internal measures, which was also emphasised as several competition levels and countries were included. Notwithstanding, another study that was not included in the previous systematic review [12] found limited variation of running and accelerometry-based measures considering playing position, stage of the season and loading during mid-week training with the exception of the two days prior to the match [13].

Furthermore, to our knowledge, only limited studies [14, 15] have attempted to quantify the TL of elite senior (first-team) and youth (U-18 team) soccer players from the same club and scarce studies are known when analysing both teams match data [16, 17]. Currently, scant literature is available comparing differences in TL between competition levels (first-team versus U-18 team) [14, 15]. This is significant as soccer players differ greatly between age groups [11–14], standards (top-class and moderate professional soccer players) [18], and the playing style of any given team [19].

In terms of developing soccer players, it may be important to understand the differences in TL between elite senior and youth players from the same club to allow practitioners to appropriately inform this process. While Buchheit et al. [20] presented physical match data of elite youth soccer players (U-13 to U-18), this study did not provide an understanding of the weekly TL throughout the season to adequately prepare these players for their match demands. Although, more recently TL comparisons have been conducted in first and youth team players [14, 15], albeit across limited periods (4 weeks) [15] and mainly examining locomotive metrics (distances covered) [14], while other accelerometry-based measures could provide impactful information for coaches. There are small discrepancies between the velocity thresholds used in the previous studies [14, 15] and those used in the general literature around soccer performance which makes a comparison of the data difficult. Specifically, the study of Houtmeyers et al. [14] found that total distance and low-intensity running (12–15 km/h) was higher for U-19 players when compared to first-team players, while distances of running at 15–20 and 20–25 km/h were similar for both teams. Moreover, first-team players covered higher running distances at > 25 km/h than U-19 players. The study of Copalle et al. [15] also showed that running distances (16–19.9 km/h, > 20 km/h and > 25 km/h) were significantly higher in U-19 players when compared to the first-team, with small effect sizes.

Examining data from different countries and leagues is vital to improving our understanding of various training methods to physically develop players [16]. Although recent research into TL in senior [11] and youth [21, 22] male soccer players have extended the existing literature, more is warranted to fully understand the different demands between young and adult players and practically apply the findings. Vigh-Larsen et al. [17] compared the U-17, U-19 and first-team from the same Danish SuperLiga club and found a higher number of accelerations and decelerations for the U-19 team when compared with both the U-17 and first-team during match-play, while no differences were found for distance covered during high-intensity running or sprinting. However, currently accelerations and decelerations have not been previously compared in youth and first-team players during training activities across consecutive seasons. Thus, it is important to quantity TL across a season and identify any differences in TL between competition levels to provide practitioners with detailed information to allow specific sessions to be designed and delivered [16].

Therefore, the aims of this study were to compare the TL among different training days and between elite senior (first-team) and youth (U-18 team) soccer players over two competitive seasons (2020–21 and 2021–2022) from an English Premiership club. Our hypothesis was that varying TL data will be evident according to the typical microcycle structure of an elite English Premiership soccer club. Moreover, based on previous studies [14, 15], it was hypothesised that first-team players will display lower loads than U-18.

## MATERIALS AND METHODS

### Participants

58 professional outfield soccer players from an English Premiership club were involved in the study. Data from the complete 2020–21 and 2021–22 seasons included 36 senior players (first-team squad) (age 23.2 ± 5.9 years, weight 75.2 ± 8.1 kg, height 1.83 ± 0.06 m), and 22 youth players (U-18 youth squad) (age 17.5 ± 1.1 years, weight 71.1 ± 8.2 kg, height 1.78 ± 0.08 m). The inclusion criteria for the study included: (i) listed on the roster of the first-team or U-18 squad of the English Premiership club at the start of the 2020–21 and/or 2021–22 seasons, (ii) regularly trained with the respective team (first-team or U-18), (iii) participated in at least 80% of training sessions and matches, (iv) did not use dietary supplements during the study, (v) who were uninjured over the course of the study, and (vi) who did not participate in another training program along with this study. Additionally, the exclusion criteria for the study included: (i) long-term injured player data, (ii) player joining the team late in either of the study seasons, (iii) lack of full, complete data for training or match-play, (iv) an in-sufficient number of satellite connection signals, and (v) goalkeepers, due to the different variations in the physical demands with outfield players.

Players were assigned to one of five positions as match demands for these differ significantly. The methodology of differentiating specialised positions was adapted from previous research [23]. As various situational factors have an influence on the style of play that can be modulated by different tactical roles [24], context was considered whilst using a player’s average position in an attempt to determine a player’s relevant tactical role in the team [25]. All participants examined were classified based on their regular playing position at the start of each season and remained consistent throughout the study period: centre-backs (CB; n = 14, senior n = 8 and youth n = 6), wing-backs (WB; n = 8, senior n = 6 and youth n = 2), centre midfielders (CM; n = 13, senior n = 9 and youth n = 4), wide midfielders (WM; n = 15, senior n = 9 and youth n = 6), and strikers (ST; n = 8, senior n = 4 and youth n = 4). Goalkeepers were excluded from the investigation due to the specific nature of their match activity and their low running demands [26, 27]. All data collected resulted from normal analytical procedures regarding player monitoring over the competitive season, nevertheless, written informed consent was obtained from all participants. Informed written consent was provided by the parents of participants under 18 years of age. The study was conducted according to the requirements of the Declaration of Helsinki and was approved by the local Ethics Committee of the University of Central Lancashire (N 0104 dated 7/12/20) and the English Premiership club from which the subjects volunteered [28]. To ensure confidentiality, all data were anonymised prior to analysis.

### Training information

Training data were collected over a two-year period across the 2020–21 and 2021–22 competitive seasons. Only team pitch-based training sessions were included for analysis. All other sessions, individual training sessions, recovery sessions, and rehabilitation training sessions were excluded [29, 30]. The planning of all soccer content was cyclical in nature and reflective of modern methods of periodisation in elite soccer and thus the external physical load experienced by players was undulating across a microcycle leading to match-play. The number of days between matches differed [31, 32] and training sessions in elite soccer microcycles have recently been classified based on days prior to a match (MD minus (-)) or post-match (MD plus (+)) [33]. All training sessions were integrated to include technical, tactical, physical and mental components. All players completed one to two strength and power gym-based sessions per microcycle incorporating upper and lower body and core exercises, although these sessions were not included in the analyses as mentioned earlier [29, 30]. All physical TL data was collected at the club’s official training facility.

Both teams only participated in one competitive league match during a microcycle and thus the structure of the training days was standardised across both teams and seasons. The first and second days post-match (MD+1 and +2) were a day off and therefore no GPS data was available. Additional fitness sessions for non-starters were limited to the immediate post-match period and GPS data was collected but not included in the study analysis. The start of the next MD microcycle was MD-4, four days prior to competition, and focussed on drills designed to develop players’ strength, power and ability to repeatedly produce explosive actions. This session was devised to improve technical and tactical understanding when ‘out-of-possession’ whilst developing the necessary physical qualities to produce high accelerations and decelerations without decrement. Individual and unit (defence, midfield, attack) practices followed by positional games and small-sided games with goalkeepers in restricted pitch dimensions were delivered. Three days pre-match (MD-3) aimed to tactically prepare players when ‘in-possession’ whilst developing position-specific high-intensity and sprint running capabilities. Practices entailed full-pitch attacking tactical patterns (10v0, 10v4) and large numbered games regularly concluding in 11v11 format (> 8v8 plus goalkeepers). The structure of MD-2, two days prior to the match, concentrated on repeating technical-tactical information at low-intensity in various functional pitch areas and dimensions and thus was regarded as an ‘under-loaded’ session considering all key GPS metrics. This session included position-specific passing patterns and then divided players into unit-specific drills for defending or attacking. The final session of the weekly microcycle, MD-1, was standardised with no variety and drills intended to provide neural stimulation to players whilst also finalising tactical situations and set-plays. For the purposes of this study, the tactical periodisation approach and subsequent TL from all MD-4, MD-3, MD-2, and MD-1 training sessions performed by both teams across the 2020–2021 and 2021–2022 seasons were standardised and examined. For the reliability and validity of the study, only data from players who performed the full session duration have been used, withdrawing the data from goalkeepers and players whose TL was manipulated due to fatigue management or injury. A total number of 493 team training sessions (first-team, n = 268; U-18, n = 225), were examined, of which 158 were MD-1 sessions (first-team, n = 88; U-18, n = 79), 126 were MD-2 sessions (first-team, n = 74; U-18, n = 52), 113 were MD-3 sessions (first-team, n = 57; U-18, n = 56), and 95 were MD-4 sessions (first-team, n = 49; U-18, n = 46). A total of 6828 individual player training session data points were included (MD-1, n = 2354; MD-2, n = 1754; MD-3, n = 1485; MD-4, n = 1235).

### Data collection

Physical data were consistently monitored across both study seasons during all training sessions and match-play using a 18 Hz Global Positioning System (GPS) technology tracking system (Apex Pod, version 4.03, 50 gr, 88 × 33 mm; Statsports; Northern Ireland, UK) that has been previously validated in a student population for tracking distance covered and peak velocity during simulated team sports and linear sprinting [34]. All devices were activated 30-minutes before data collection to allow the acquisition of satellite signals and to synchronise the GPS clock with the satellite’s atomic clock [35]. Quantifying the devices’ accuracy indicated a 2.5% estimation error in distance covered, with accuracy improving as the distance covered increased and the speed of movement decreased [36]. To avoid inter-unit error, each player wore the same device during the study period [37, 38], although the present GPS system has previously reported excellent inter-unit reliability [39]. Specifically designed vests were used to hold the devices, located on the player’s upper torso, and anatomically adjusted to each player, as previously described [40]. To avoid potential inter-unit variation players wore the same GPS unit for each training session and match [40]. The GPS signal quality and horizontal dilution of position was connected to a mean number of 21 ± 3 satellites, range 18–23, while HDOP for both seasons was 0.9 (first-team) and 1.3 (U-18), respectively. On completion of each session, GPS data were extracted using proprietary software (Apex, 10 Hz version 4.3.8, Statsports Software; Northern Ireland, UK) as software-derived data is a more simple and efficient way for practitioners to obtain data in an applied environment, with no differences reported between processing methods (software-derived to raw processed) [41]. The dwell time (minimum effort duration) was set at 0.5 s to detect high-intensity running and 1 s to detect sprint distance efforts, in-line with manufacturers recommendations and default settings to maintain consistent data processing [40]. Furthermore, the internal processing of the GPS units utilised the Doppler shift method to calculate both distance and velocity data which is shown to display a higher level of precision and less error compared with data calculated via positional differentiation [42]. Statsports provided written permission to allow all data to be used for research purposes.

The total distance covered (m); high-intensity distance (m; total distance covered 5.5–7 m · s^−1^); sprint distance (m; total distance covered > 7 m · s^−1^ were examined and have been established based on previous studies [31, 43]. The following physical variables were also quantified in this study: explosive distance (m; distance covered with acceleration above 1.12 m/s^−2^); the number of very high-intensity accelerations (> +3 m · s^−2^ with minimum duration of 0.5 s); the number of very high-intensity decelerations (< -3 m · s^−2^ with minimum duration of 0.5 s) [13, 37].

### Statistical analyses

Data are displayed as the mean ± standard deviation. Linear mixed-effect models with random intercepts for individual players’ ID were used to assess the effects of the team (first-team / U-18), training day (MD-4, MD-3, MD-2, MD-1), and their interaction on the examined GPS metrics. When there was a significant effect of the team and/or training day and/or their interaction, post-hoc Tukey’s HSD tests for pairwise comparisons were performed to examine which categories differed and determine the source of the interaction. The Cohen’s d effect-size (ES) statistic was calculated to determine the magnitude of effects by the difference of two population means which are then divided by the standard deviation from the data. Absolute differences between teams were standardised by the respective between-player standard deviation of each outcome variable to determine an effect size (ES). Standardised differences were evaluated as trivial (< 0.2), small (0.2–0.6), moderate (0.6–1.2), large (1.2–2.0), very large (2.0–4.0), and extremely large (> 4.0) [38]. The statistical analyses were performed in R language and environment for statistical computing (version 4.2.0, The R Foundation for Statistical Computing, Vienna, Austria), using the packages nlme and lsmeans [44]. For all analyses, statistical significance was set at p < 0.05.

## RESULTS

**FIG. 1:**
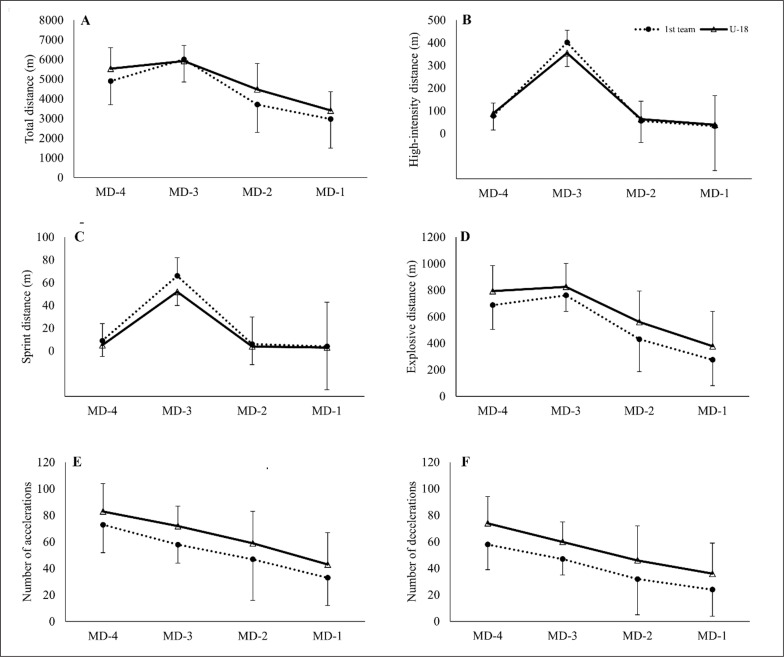
Mean and standard deviation values of total distance (A), high-intensity distance (B), sprint distance (C), explosive distance (D), the number of accelerations (E), and the number of decelerations (F) across the training microcycle from the two examined teams. The dotted line with black circles: first-team; solid line with white triangles: U-18.

Figure 1 shows the mean and standard deviation values of the examined GPS metrics from both teams across the MD-4 to MD-1 training days.

The linear mixed-effect model analyses revealed a significant (p < 0.01) main effect of the team for all examined variables apart from high-intensity distance (p = 0.081). Irrespective of the training day, when compared to first-team players, U-18 players covered greater total and explosive distance, and performed a higher number of accelerations and decelerations. Conversely, first-team players covered greater sprint distance than U-18 players. Also, a significant (p < 0.01) main effect of the training day was detected for all examined variables. Post-hoc pairwise comparisons for the training day revealed that, irrespective of team (first-team / U-18), the total distance covered, high-intensity distance, sprint distance and explosive distance were greater on MD-3, followed by MD-4, MD-2 and MD-1. The above differences between days were significant (p < 0.05) for all examined metrics and for all pairwise comparisons between any pair of training days, with the exception of MD-2 vs. MD-1 comparison for sprint distance (p = 0.259). Conversely, the number of accelerations and decelerations was higher at MD-4, followed by MD-3, MD-2, and MD-1. For both accelerations and decelerations, the differences between all training days were statistically significant (p < 0.05), that is, MD-4 > MD-3 > MD-2 > MD-1. The team-by-training day interaction was significant (p < 0.01) for all examined outcome variables. To explore the source of the interaction, pairwise comparisons between teams at any given training day were examined as post-hoc analysis after the linear mixed-effect model analyses were conducted. Table 1 displays the estimated differences between teams for any given training day.

**TABLE 1:** Estimated differences between teams for the examined metrics on all training days. CI: confidence interval; ES: effect size

		First-team / U-18 estimated difference (95% CI)	p value	ES
**Total Distance (m)**	**MD-4**	-435 (-696 to -173)	< 0.001	1.08
	**MD-3**	+279 (+27 to +532)	0.018	0.70
	**MD-2**	-590 (-842 to -339)	< 0.001	1.47
	**MD-1**	-235 (-467 to -4)	0.043	0.59

**High-intensity distance (m)**	**MD-4**	-7 (-30 to +15)	0.976	0.33
	**MD-3**	49 (28 to +71)	< 0.001	2.23
	**MD-2**	-6 (-27 to +16)	0.993	0.26
	**MD-1**	-3 (-23 to +17)	1.000	0.14

**Sprint distance (m)**	**MD-4**	+5 (0 to +11)	0.047	1.00
	**MD-3**	+15 (+10 to +20)	< 0.001	2.84
	**MD-2**	+4 (-1 to +9)	0.279	0.73
	**MD-1**	+2 (-3 to +7)	0.885	0.38

**Explosive distance (m)**	**MD-4**	-36 (-84 to +11)	0.278	0.42
	**MD-3**	+5 (-41 to +52)	1.000	0.06
	**MD-2**	-58 (-104 to -12)	0.004	0.66
	**MD-1**	-29 (-72 to +14)	0.431	0.34

**Number of accelerations**	**MD-4**	-5 (-10 to 0)	0.103	0.47
	**MD-3**	-9 (-14 to -4)	< 0.001	0.91
	**MD-2**	-6 (-11 to -1)	0.006	0.61
	**MD-1**	-4 (-8 to +1)	0.305	0.35

**Number of decelerations**	**MD-4**	-6 (-11 to -1)	0.003	0.56
	**MD-3**	-2 (-7 to +2)	0.821	0.21
	**MD-2**	-4 (-9 to +1)	0.171	0.36
	**MD-1**	-1 (-6 to +3)	0.992	0.11

U-18 players covered greater total distance than first-team players at MD-4, MD-2, MD-1, with small (ES = 0.59) to large (ES = 1.47) differences. On the contrary, at MD-3, first-team players covered a greater total distance than U-18 players, with a moderate difference (ES = 0.70). For high-intensity distance, the differences between teams were not statistically significant at MD-4, MD-2, and MD-1, while first-team players covered greater high-intensity distance than U-18 players at MD-3, with a very large difference (ES = 2.23) between teams. The sprint distance covered was greater for the first-team compared to U-18 players at MD-4 with a moderate difference (ES = 1.00), and at MD-3 with a very large difference (ES = 2.84), while there were no significant differences between teams at MD-2 and MD-1. Explosive distance was greater for U-18 players than for first-team players at MD-2, with a moderate difference (ES = 0.66), whereas no significant differences were detected at MD-4, MD-3, and MD-1. Finally, U-18 players performed a higher number of accelerations than first-team players at MD-3 and MD-2 (both moderate differences, ES = 0.91 and 0.61, respectively), and a higher number of decelerations at MD-4, with a small difference (ES = 0.56). On all other training days, no significant differences were observed between teams for the number of accelerations and decelerations (Table 1).

## DISCUSSION

The aims of this study were to compare the TL among different training days and between elite senior (first-team) and youth (U-18 team) soccer players over two competitive seasons (2020-21 and 2021-2022) from an English Premiership club. Regardless of the team, the main results showed that the high-intensity distance, sprint distance and explosive distance were greater at MD-3, followed by MD-4, MD-2 and MD-1. Moreover, the number of accelerations and decelerations was higher at MD-4, followed by MD-3, MD-2, and MD-1. When comparing both teams, total distance was greater for U-18 players at MD-4, MD-2, MD-1, and greater for the first-team at MD-3. While, high-intensity and sprint distance were greater for the first-team at MD-3 (sprint distance also slightly greater at MD-4) and accelerations and decelerations were higher for the U-18 team.

To the best of the authors knowledge, this was the first study that analysed training data from two different age group teams from the same English Premiership club that contributes additional new findings for the specific context of English Premiership soccer. One of the strengths of the present study was that data from two consecutive seasons were used with the aim of data becoming more robust and not comparing data between seasons, where different players and coaches were used in both seasons, which was not the current scenario. The other major strength was the comparison of two teams, U-18 and first-team, which is very scarce in the literature [14, 15].

Nonetheless, considering the range values previously presented in the systematic review of young soccer players [22], the present data found that the U-18 team was within range for total distance of 3964–6500 m, greater for high-intensity distance (although with a different threshold) (12–250 m) and also greater for sprint distance (0–30 m) [22]. Regarding the number of accelerations and decelerations, the values seemed to be similar to those displayed by U-17 and U-19 Portuguese soccer players [45] with a slight tendency of higher values for the U-18 team of the present study. Moreover, considering the range values presented in the previous systematic review in professional soccer players, namely, total distance 2143–9540 m, distance > 18 km/h = 7–541 m, distance > 24 km/h = 1–190 m, acceleration number > 3 m · s^−2^ = 9–195, deceleration number > -3 m · s^−2^ = 10–157 [10], the findings of this study showed that the first-team values were within previous ranges.

Regarding the findings related to higher values at MD-3, followed by MD-4, MD-2 and MD-1 for total distance, this was similar to a previous study in U-18 English Premier league players that also found the second training day of the week produced higher values for running distance variables [46]. Similar findings in U-18 Spanish players for running, high-intensity and sprint distance revealed higher values at MD-3 and MD-2 when compared with the other training sessions, although only six training sessions from non-identical microcycle structures were reported [47]. Additionally, the present study found higher values at MD-4 for accelerometry-based variables. Still, no studies were found that analysed microcycles with only four training sessions in U-18 soccer players, although with three training sessions, it has been previously demonstrated that higher values for both accelerations and decelerations in the first training session of the week occur, while running distances were higher on the second training session of the week [45], which is similar to the findings of the present study. A study [13] in U-18 players with only five training sessions showed limited variation between MD-4, MD-3 and MD-2 which opposes the present study findings. Furthermore, other studies that included data from non-identical microcycle structures (which provided six training sessions) showed that accelerations and decelerations were higher at MD-4 compared with other training sessions in U-18 players [47].

In the professional first-team players analysed in the current study, the same pattern was observed, which was corroborated by the range values highlighted in the previous systematic review [11]. Indeed, previous studies with four training sessions showed different results. For instance, higher values at MD-3 (or the second session of the week) for running distances and accelerometry-based variables were also reported by English Premier League players [33] and for sprinting by Dutch Eredivisie players [32]. Nonetheless, higher values were shown at MD-4 (or the first session of the week), for running distances by Portuguese Premier League players [48] and for both running and accelerometry-based variables in Dutch Eredivisie players [32]. A possible justification for some differences between the results of the present study and the previous literature could be attributed to the different training competitions [46] and different coaching philosophies and training methods [49].

Regarding the comparison between the first and U-18 teams, it was observed that the first-team covered greater high-intensity and sprint distance especially at MD-3 compared with U-18 players, while the other training days were similar. This may be attributed to the use of absolute speed thresholds [50]. Even so, the present data suggests that first-team team players have greater sprint capabilities than youth players. Thus, future studies should test individual thresholds with English Premiership players. Additionally, at MD-4, MD-2 and MD-1, the U-18 team covered a greater total distance which may be associated with less competitive pressure compared to the first-team environment. A study conducted in the Chinese Super league observed that some positions such as central defenders and fullbacks covered more high-intensity and sprint running distance in the high possession teams, while wide midfielders and forwards covered more high-intensity and sprint running distance in the low possession teams [51]. Although the context of high/low ball possession was not considered in the present research, it is possible that it could justify the present results. Thus, it is suggested to confirm such possibility in future investigations. Even so, there may be a practical application suggestion to develop physical capacities such as aerobic fitness, especially on MD-4 with on-pitch training, without the concern of an undulating, tapering strategy to recover players for the forthcoming match. Furthermore, the U-18 team performed more explosive distance, a greater number of accelerations and decelerations which may be attributed to the different training drills implemented and again a variation of a tapering strategy when compared with the first-team.

Despite the findings of this study, there are some limitations that should be listed: a) only one youth team and one professional soccer team players were used which consequently avoided the analysis of playing positions due to the small number in each team; b) no variable of internal load was used which could strengthen the findings of this study; c) generalisation of the results should be cautious as both teams analysed belonged to the same English Premiership club which may be different in other leagues and countries; d) any positional change across the two seasons, during the season or within weekly match-play that would alter the match demands for individual players was not considered; and e) other contextual factors such as formation change or change of manager and style of play that would also influence physical match demands was not measured.

Future studies should consider a study design that may include the analysis of starters and non-starters, with special regard to the first training session after the match, the analysis of playing positions, and the analysis of contextual variables such as the number of the matches in the week, match results, match location, and quality of opponents. For instance, a recent study on professional soccer players showed that match location, match outcome and level of the opponent slightly affected the weekly external TL while playing positions showed several differences [52]. Furthermore, when considering the number of matches, Clemente et al. [48] showed that acute load and training strain presented higher for players that started two or three matches in the same week. Finally, the inclusion of simultaneous match and TL data would provide greater insights and allow further analysis (e.g. training/match ratios [53]) into youth and first-teams.

## CONCLUSIONS

In summary, we observed higher values in some of the selected training-related variables at MD-3 in two teams from the same English Premiership soccer club during the second training session of the week, while there were also higher values of different metrics (accelerations and decelerations) in the first training session of the week (MD-4). Specifically, U-18 players covered a higher total distance in the majority of training sessions (MD-4, MD-2, MD-1). Moreover, they also performed a higher number of accelerations and decelerations in all training sessions when compared with the first-team. Nonetheless, first-team players covered greater values of high-intensity and sprint distances at MD-3 (sprint distance also slightly greater at MD-4) than U-18 players. To our knowledge, the main strength of this study is the comparison between young and adult soccer players in the context of the same club over an extended period (two full consecutive seasons).

### Practical applications

The current study provides information regarding the microcycle periodisation, and the type of exercises used in the training sessions. In addition, it provides average values that can be applied by other teams and coaches from similar contexts. Moreover, it shows that to train both youth and first-team players, TL may vary in terms of intensity and that different types or exercise choices and contextual competition may be of additional importance when preparing the microcyle plan. This study showed that U-18 soccer players are prepared to cope with first-team TL demands in terms of total distance, accelerations, and decelerations. Nonetheless, considering high-intensity, sprint and explosive distances, it would be better to increase such values before progressing to the first-team, although from a statistical point of view, such differences were almost non-existent.
